# Research on Dynamic Evolution Model and Method of Communication Network Based on Real War Game

**DOI:** 10.3390/e23040487

**Published:** 2021-04-20

**Authors:** Tongliang Lu, Kai Chen, Yan Zhang, Qiling Deng

**Affiliations:** 1 College of System Engineering, National University of Defense Technology, Changsha 410073, China; lutongliang@nudt.edu.cn (T.L.); dengqiling18@nudt.edu.cn (Q.D.); 2 College of Information and Communication, National University of Defense Technology, Wuhan 430014, China; M201773001@alumni.hust.edu.cn

**Keywords:** combat System-of-Systems, generation algorithm, dynamic network evolution

## Abstract

Based on the data in real combat games, the combat System-of-Systems is usually composed of a large number of armed equipment platforms (or systems) and a reasonable communication network to connect mutually independent weapons and equipment platforms to achieve tasks such as information collection, sharing, and collaborative processing. However, the generation algorithm of the combat system in the existing research is too simple and not suitable for reality. To overcome this problem, this paper proposes a communication network generation algorithm by adopting the joint distribution strategy of power law distribution and Poisson distribution to model the communication network. The simulation method is used to study the operation under continuous attack on communication nodes. The comprehensive experimental results of the dynamic evolution of the combat network in the battle scene verify the rationality and effectiveness of the communication network construction.

## 1. Introduction

Modern warfare is a confrontation between systems. The result of a war depends not only on the performance of the equipment but also on the structure of the combat system. The combat system includes all command and control (C2) units, sensor units, attack units, and communication units. There are complex multi-layer connections between each unit. If taking all units into nodes, and taking the connection relationship into edges, then the combat system will become a combat network. From the topological structure, the combat system can be regarded as a combat system consisting of an attack entity, a command entity, a sensor entity, and a communication entity. Nodes constitute a network formed by the interaction of energy flow, information flow, and material flow. The existing combat platforms and weapon systems basically have functions such as data transmission, information monitoring, and remote control. Among them, the combat system is composed of a large number of combat platforms or systems, and a reasonable communication network must be established to achieve the combat system with integration of energy flow, information flow, and material flow. The combat system connects independent weapon platforms through a communication network to collect, process, distribute, and transmit battlefield information in real time, thereby forming the overall emergence of the combat system.

With the development of new combat forms and new weapons and equipments employing, the communications network is not only an important part of the combat System-of-Systems (SoS), but it also can be regarded as a type of weapon system with the characteristics of the systems. There is still a lack of systematic research on the construction and evaluation methods of the evaluation index system of the communication network system. In the future, who predominates the information will win the war. The importance of communication networks in the war has gradually become prominent, which also puts forward new requirements for the construction of communication networks in the future war.

In the 19th century, C.V. Clausewitz [1] proposed the “fog of war” to describe the characteristics of war. In today’s high-tech wars involving five-dimensional operations land, sea, air, space, and electromagnetic are full of uncertain factors. Electromagnetic is considered to be the “fifth-dimensional battlefield” after land, sea, air, and space, which mainly refers to radio interference, electromagnetic pulse attacks and protection. At the beginning of the twentieth century, F.W. Lanchester [2] proposed a set of differential equations describing the relationship between the forces of the two sides during the battle and also used linear and square laws to quantify the relationship between cold and hot weapons. Between 1965 and 1996, C.J. Ancker, M. Kress, and S. Parkhideh et al. [3] conducted in-depth research using probability and random process theory. At the end of the 20th century, J.H. Holland [4] put forward the complex theory, D.S. Albert, J.J. Garstka, and F.P. Stein [5] published the famous monograph: network-centric warfare. War modeling based on complex systems and complex networks has become the latest research field. Combat platforms (entities) are divided into nodes and different flow such as material flow and energy flow.

**Model structure.** The classical static networks loop model of J. R. Cares [6] distinguishes all nodes into four classes: sensors, deciders, influencers, and targets, and it uses the eigenvalue of the adjacency matrix to evaluate the efficiency of the operation network. An Information Age Combat Model is introduced, and the mathematics of its structure, dynamics, and operational evolution are presented; policy, doctrine, and acquisition implications are explored; and a tutorial of relevant mathematics is appended. D. F. Tan et al. [7] advance the research on the networks loop model by researching the common structure of a network of a combat system, which consists of reconnaissance forces, a command department, attack forces, and target connected together by a reconnaissance network, command and control network, and engagement network. They provide a deeper analysis of Cares’ efficiency evaluation model based on an adjacency matrix and indicate two defects of Cares’ model [8]: first, a static model cannot reflect the dynamic changes of network structure during the operation; second, many nodes may belong to more than one classes, for example, fighter planes can be sensors, influencers, and even deciders, and most nodes can possibly be targets of enemy. A. H. Dekker [9] compares networks with different topology structures when they combat with large numbers of decentralized agents. The finc force, intelligence, networking, and C2 methodology allows the calculation of three metrics for every C4ISR architecture. He uses the percentage of destroyed decentralized agents to evaluate the efficiencies of these works and also compares the robustness of networks with different topology structure when they are under random and deliberate attack. Dekker [10] put forth his research on centralization and self-synchronization of multi-agent systems, turning his interest to self-synchronization in C2 networks. Two important recent trends in military and civilian communications have been the increasing tendency to base operations around an internal network and the increasing threats to communications infrastructure.

**System modeling.** J. Moffat [11] proposed the concept of war from complex network theory and constructed the mathematical model, knowledge, and conflict of war games. Edward A. Smith analyzed the complexity of war, and he believed that effect-based attack methods and networks are the key to victory. Hu Xiaofeng et al. [12,13] conducted in-depth research on the combat system using complex networks, constructed the network topology of the combat system, analyzed the command hierarchy and command mechanism of the combat system based on simulation data, and adjusted the command nodes and communications in the combat system network. By adjusting the changes in the different ratios between the command and communication nodes in the combat system network, he studied the impact on network distribution. Tan Yuejin [14] made further improvements on the basis of J.R. Cares [15] research, abstracted weapons and equipment as nodes and connections as edges, established a combat network model, and divided the equipment into reconnaissance, C2, and influence functions according to the functions of weapons and equipment. The node abstracts the equipment system as a combat network. The combat ring is used as an index to evaluate the combat effectiveness of the weapon equipment system. Chen Lei et al. [16] analyzed the statistical characteristics of the combat system network based on real combat games but did not provide a generalizable model and generation algorithm, which could not guide the rational construction of the network topology on fighting in the future.

**Network topology.** The current large-scale combat system modeling research based on complex network theory lacks a generation algorithm that conforms to the reality of the war, which cannot provide basis and support for further research on the dynamic evolution of combat networks and the evaluation of combat SoS in confrontation.

Complex network theory uses topology as a starting point to study the structural characteristics of networks. Therefore, studying the fragility and robustness of combat networks is a hot topic for many scholars. The most common method is to describe through global and local indicators. The global indicators include several common quality assurance such as maximum connectivity, connectivity factor, load loss rate, etc. In the literature [17], the maximum connectivity is used as an indicator to study the similar characteristics of the power information–physical dependency network and verify that the low degree nodes increase the strategy of connecting edges.

The Information Age Combat Model (IACM) [18] proposed by Cares adopts a network structure and uses nodes to represent the energy flow or information flow between entities. The interactions between the four typical types of nodes constitute a complete Observe–Orient–Decide–Act (OODA) cycle. The network loop is the source of combat effectiveness. The Perron–Frobenius of the IACM network adjacency matrix is proposed as an indicator to measure the robustness, indicating that there is a close relationship between this indicator and the effectiveness of the system’s combat effectiveness; Sean et al. [18] further the rationality of the PFE index, which was verified by simulation. However, there are obvious shortcomings: the network ring can only reflect the influence of the network structure of the combat system on the robustness of the OODA cycle [19], and the influence of the component system capabilities is ignored; at the same time, the PFE can only represent the number of network rings in the combat system network. It cannot reflect the number of effective networks that exert actual combat effectiveness.

Bong Gu Kang [20,21] proposed the communication system in the network-centric warfare (NCW) has been analyzed from the perspective of the SoS, which consists of a combat system and a network system so that the two reflect each other’s effects. Then, he put forward simulation of a SoS model, which consists of a combat model and a network model, which has been used to analyze the performance of network-centric warfare in detail. The main contributions of this paper are as follows:

(1) Overcoming the disadvantage of the generation algorithm in the traditional combat SoS, and using the joint distribution strategy of power law distribution and Poisson distribution to model the communication network;
(2) Using simulation methods to study the changes in the combat network under continuous attacks on communication nodes;
(3) Using the method of control experiment to verify the rationality and effectiveness of the communication network modeling, which provides a reference value for the construction of the communication network topology in war.

## 2. Generation Algorithm

We based our research on empirical data from a real war game for which we built the complex network model of real combat SoS. This war game is designed to be a SoS counterwork between the Red force and the Blue force. The Red force needs to palsy the combat SoS of the Blue force and preserve the combat SoS of itself simultaneously. These data do not include detailed parameters of any platform in the war game. However, it records the development of the network structure of the Red force combat SoS, which allow us to do a lot of significant work on it.

**Figure 1:**
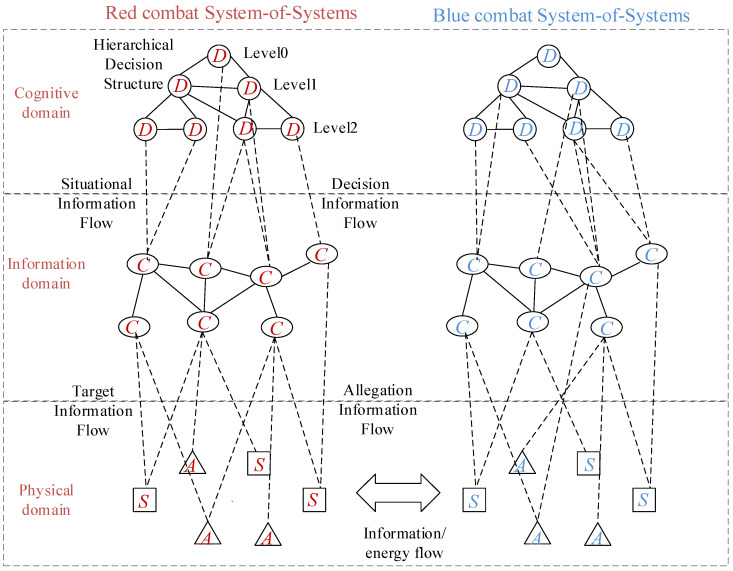
The diagram of relational hierarchy of the network structure of the combat SoS.

**Figure A1:**
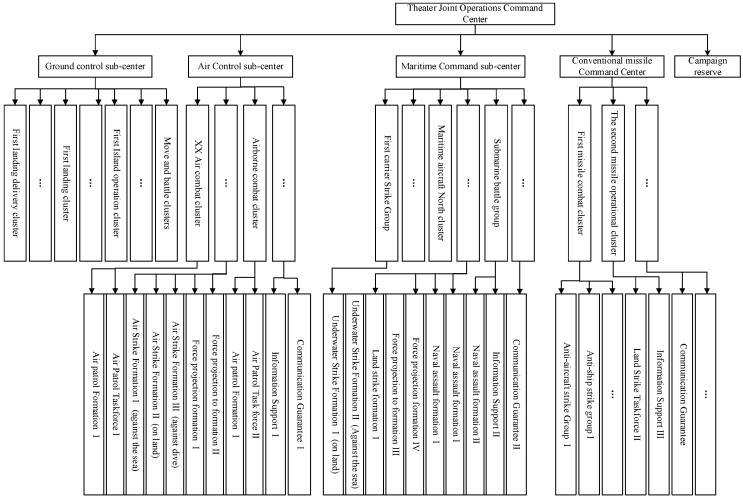
The relationship between the sea and air joint strike command organization.

Similar to the static network loop model of J.R. Cares, we distinguish all operation platform (nodes) in the war-game into four classes: C2 nodes, communication nodes, sensors, and attack nodes. The diagram of relational hierarchy of the network structure of the Red and Blue force combat SoS is shown in Figure 1, where 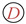 denotes the C2 node, 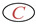 denotes the communication node, 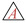 denotes the attack node, and 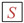 denotes the sensors node. We can see that the communication network acts as the combination [22] of the C2 subnetwork, sensors subnetwork, and attack subnetwork, and the multifunctional nodes, such as some fighter aircraft, which could be both sensors and attackers and sensors, act as the joint nodes of each subnetwork. The relationship between the sea and air joint strike command organization is shown in Figure A1.

It can be seen that the C2 subnetwork is like the head of the whole combat SoS; it is directly connected with the lowest level hub nodes in the communication network, which establishes the connection relationship with the sensors subnetwork and the attackers subnetwork, thus having an impact on the entire combat system [23]. As a bridge connecting the entire combat network, the communication node has an important role and significance.

**Figure 2:**
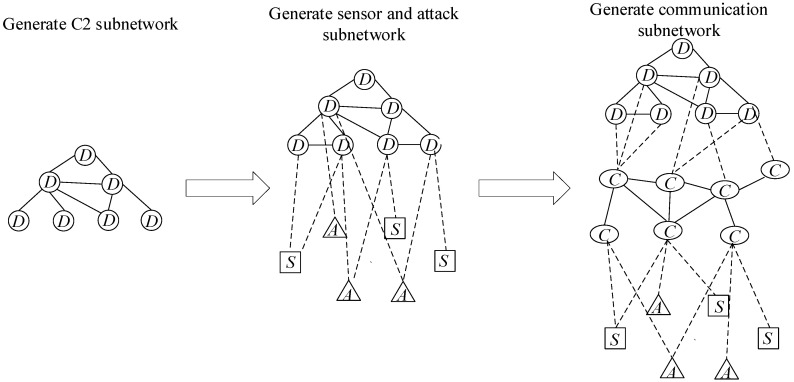
The generation of the combat network.

As shown in Figure 2, the network is constructed in a hierarchical structure. First, we generate the C2 subnetwork. Then, we generate the sensor and attack subnetwork. Finally, the communication network is used to replace the connection between C2 nodes, sensor nodes, and attack nodes. Compared with the direct connection between C2 nodes, sensor nodes, and attack nodes, the generated communication network can satisfy the communication between the C2 subnetwork and the sensors and attack subnetwork. At the same time, increased network redundancy can improve the communication efficiency and survivability of the network.

### 2.1. C2 Subnetwork

As we can see from the structure in Figure 1, the combat network is divided into three levels, the structure shows that C2 subnetwork is similar to the head of whole combat SoS. Table A1 in Appendix A shows the details of all C2 nodes, and Figure 2 shows the initial structure of the C2 subnetwork. Node in the C2 subnetwork is shown in Table A1.

The level-0 node plays the most important role in the reconfiguration. On one hand, the level-1 node act as a real-time backup of the level-0 node, which ensure conflict will happen, and the operation command could be continued when the level-0 node is destroyed; on the other hand, the level-1 nodes are empowered to take over the duty of level-2 nodes and any third-level command center when they are destroyed. Therefore, the robustness [24] of the C2 subnetwork is significant. All C2 nodes in the fourth-level have no constant entities, their work is done by subordinate nodes, which have the maximum degree.

C2 subnetwork generation algorithm:

Specify the C2 network level number L and specify the C2 level node number array $\left[N_{1},N_{2},\ldots N_{L}\right]$, where $N_{L}$ is the central C2 node number and $N_{L}$ is the outer C2 node number.

(1) Generate the first level of C2 nodes, the number of C2 nodes of the first level is $N_{1}$, and generate the central node (root node $D_{0}$), which is also the highest level C2 node, the number is 1; set the level number flag h=1;
(2) The generation of second-level $D_{i},i<N_{2},N_{2}$ nodes is the number of nodes, the second-level command nodes are connected to the central command node, and the same-level command nodes are interconnected according to probability $P_{d}$ to form a command coordination relationship.
(3) The outer command nodes are generated in sequence, and the level number flag h=h+1 is set. The command nodes of the same layer are interconnected according to probability $P_{d}$ to form a command coordination relationship, and according to probability $P_{C2}$, they are connected with non-directly connected upper-level nodes to form a cross-layer command relationship.
(4) Repeat step (4) until the C2 network is generated.

### 2.2. Sensors and Attack Subnetwork

The real connection strategy determines the attack and sensor nodes; that is, the outermost C2 nodes of the C2 network belong to the unit of the combat formation, which is not separated from other attributes of the formation, such as attack or sensor attributes. Therefore, the leaf nodes of the C2 network are connected with at most one attack and sensor node. Nodes in the attack and sensors subnetwork are shown in Table A2.

Specify the number of sensor nodes as $S_{n}$ and satisfy $S_{n}<N_{L}$. We randomly select $S_{n}$ sensor nodes from $N_{L}$ C2 nodes, and each C2 node $D_{i}$ generates a corresponding sensor node $S_{i}$, where $i<S_{n}$. The sensor node degree is distributed according to the power law. The maximum weight of the sensor node is defined as W. The Poisson distribution curve is fitted according to the weight parameter, and the connection probability P of each sensor node is generated. The sensor node self-connects according to the probability P to form a sensor subnetwork. Similarity, we can generate the attack subnetwork. Specify the number of attack nodes as $F_{n}$, which satisfy $F_{n}<N_{L}$. We randomly select $F_{n}$ attack nodes from $N_{L}$ C2 nodes, and each C2 node $D_{i}$ generates a corresponding attack node $F_{i}$, among which $i<F_{n}$.

The attack node is at the edge of the combat SoS and belongs to the leaf node, receiving the information and instructions from sensor nodes and C2 nodes to complete relevant combat tasks. Targeted information and C2 information flow through the attack node, comprehensively using various types of weapons and equipment or methods of fighting to attack enemy targets. The attack node is at the edge of the combat network; as a combat unit, it usually has the attributes and functions of sensors, communication, C2, and attack, such as the air patrol formation and ground attack formation. Therefore, here, we are no longer able to distinguish the sub-attributes of the attack network separately but rather, we intend to define the granularity of the study as a combat unit and analyze the relationship between the fusion of its internal multiple attributes and other types of nodes in the network.

Here, the attack node is the soft kill and hard kill weapon equipment (platform or system) that affects the enemy, which is collectively called the attack node. Therefore, the attack node can understand weapons and equipment entities that can destroy and interfere with the enemy. Destruction refers to a means to cause damage or loss of combat capability to the enemy within a certain time and scope, while interference is a means to instruct the enemy’s electronic systems and equipment losing their original capability.

### 2.3. Communication Subnetwork

According to the related theory of a multi-layer interdependent network, the C2 subnetwork, the sensor subnetwork, and the attack subnetwork are self-connected and interconnected through the communication subnetwork by defining the model as a three-layer dependent network. The interdependent network is fitted according to the joint distribution [25] of power law distribution and Poisson distribution. Nodes in the communication subnetwork is shown in Table A3.

Relying on the real situation, the communication subnetwork is divided into a two-layer network. The inner communication network is mainly responsible for the communication between the C2 networks, and the outer communication network is responsible for the communication between the attack and sensor subnetwork. Specify the number of two-layer communication $\left[N_{co},N_{c1}\right]$ relay network nodes and generate communication relay nodes $C0_{i},C1_{j}$ respectively, where $i<N_{CO},j<N_{C1}$.

Define the three connection types of each communication relay node, expressed as *x*, *y*, *z*, where *x* is the degree of dependence of the communication subnetwork on the C2 network, *y* is the degree of dependence of the communication subnetwork on the sensing and C2 network, and *z* is the communication degree of self-connection.

Define joint distribution function:

(1) $$F_{COM}^{Q}\left(x,y,z\right)=\lambda F_{COM}^{BQ}\left(x,y,z\right)+\left(1-\lambda \right)F_{COM}^{FQ}\left(x,y,z\right)$$

where λ represents the mixing rate [26] of the two types of distributions, and x,y,z respectively represents the degree of dependence of the communication subnetwork on the C2 network, sensors network, attack network, and the communication network. $F_{COM}^{BQ}$ represents Poisson distribution, simulation generation uses the ER random network generation algorithm, $F_{COM}^{FQ}$ represents power-law distribution, and simulation generation uses the BA growth network generation algorithm [27].

Traversing the edges of the C2 network, the two C2 nodes of the edge select the inner communication relay node to connect according to the joint distribution function, and they complete the self-connection of the inner subnetwork at the same time.

Traversing the edges of the attack and sensor networks, the two endpoints of the edge select the outer communication relay node to connect according to the joint distribution probability function, and they complete the self-connection of the outer communication relay node.

Traversing the edges between the outermost C2 and sensor network, according to the joint distribution probability function, the C2 nodes select the inner communication relay nodes, and the sensor nodes select the outer communication relay node to connect and complete at the same time the self-connection of internal and external communication relays.

Cancel the connection of the original C2 subnetwork, sensor subnetwork, and attack subnetwork to form the final three-layer dependent communication network topology.

### 2.4. Network Topology and Degree Distribution Analysis

**Figure 3:**
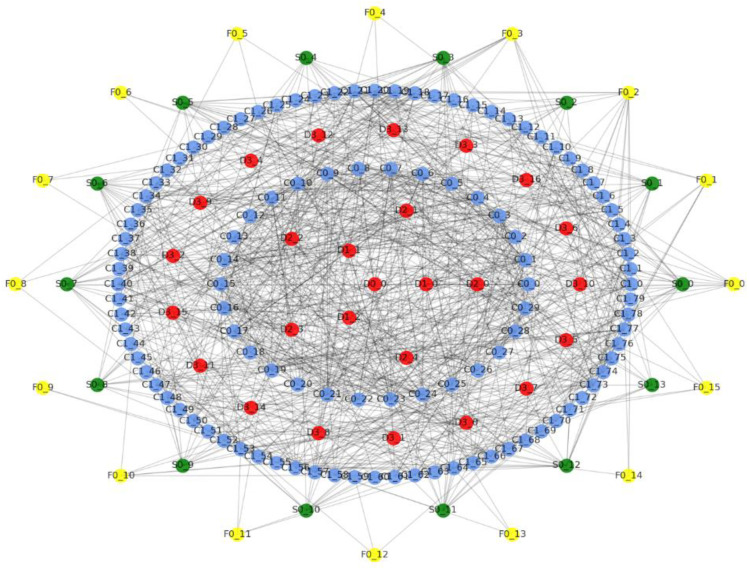
Hierarchical diagram of combat network topology.

From the topological structure of the combat network, it can be seen that the combat network is central and hierarchical. The outermost leaf nodes are sensor nodes and attack nodes, the central node is the commander node, and the communication node connects the central node. Among them, the communication processing node is at the top C2 node level, and the outermost task formation (the 4th level C2 node) has no entity C2 nodes with only C2 attributes, and the formations are not interconnected. The sensor network is sorted according to the importance of the sensor nodes; then, weights are obtained according to the power-law distribution, and they are self-connected according to the probability. The attack subnetworks are not interconnected. The interconnection probability [28] of the sensor nodes and attack nodes is sorted according to the importance of the attack nodes, and then the weights are obtained according to the power law distribution and then interconnected according to the probability. Sensor nodes and non-affiliated command nodes are connected according to probability. The modeling combat network is shown in the Figure 3.

**Figure 4:**
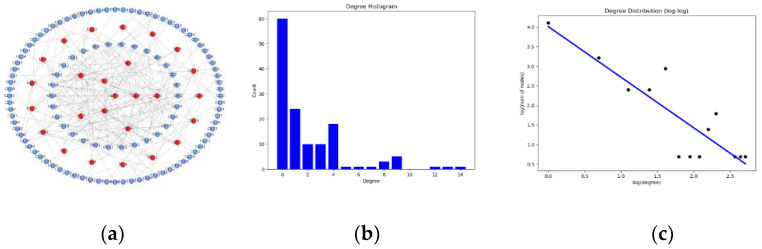
C2 subnetwork degree distribution. (**a**) The network topology. (**b**). Histogram of the node digrees of C2 subnetwork. (**c**) Log distribution of the node digrees of C2 subnetwork.

It can be seen from Figure 4 that the degree distribution of the C2 subnetwork presents an obvious power-law distribution. In the actual combat network, the C2 network plays an important role such as information receiving, processing, coordination, uploading and distributing. Among them, (1) there is a distinction between superior and subordinate; and (2) there is a cooperative relationship of information transmission in the same level of the C2 subnetwork. Therefore, the characteristics of the C2 network can be regarded as a multi-layer network [26] formed by coupling the WS small world network and the BA scale-free network.

**Figure 5:**
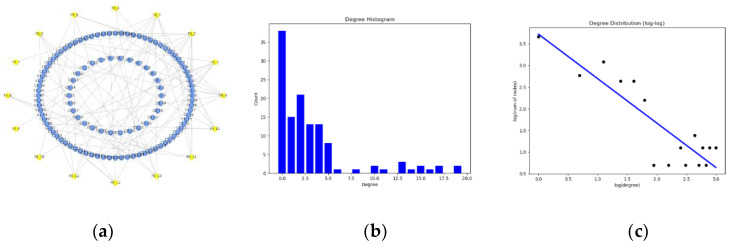
Sensors subnetwork degree distribution. (**a**) The network topology. (**b**). Histogram of the node digrees of sensors subnetwork. (**c**) Log distribution of the node digrees of sensors subnetwork.

**Figure 6:**
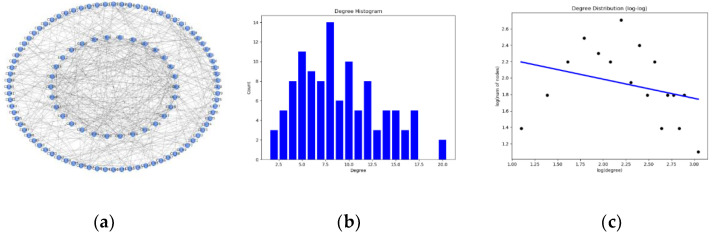
Degree distribution of the communication subnetwork. (**a**) The network topology. (**b**). Histogram of the node digrees of communication subnetwork. (**c**) Log distribution of the node digrees of communication subnetwork.

It can be seen from Figure 5 that the sensor subnetwork is an open network with strong perception and interaction capabilities for the external environment. The communication network directly transmits the information obtained by the sensors to the C2 nodes to realize situational awareness. We use python’s distribution fitting tool to realize the degree distribution statistics of the sensor subnetwork, and the result is shown in Figure 6. Obviously, the degree distribution of the sensor subnetwork is not linear, indicating that the sensor subnetwork has a certain degree of modularity: the sensor network is a flat network with community characteristics, which is of great significance to the improvement of the efficiency of information transmission between networks. Sensor nodes can quickly join and exit, which reduces the failure rate of the network and promotes the information fusion of the entire combat network.

**Figure 7:**
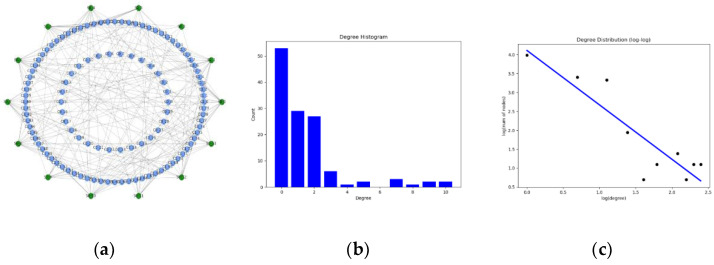
Attack subnetwork degree distribution. (**a**) The network topology. (**b**). Histogram of the node digrees of attack subnetwork. (**c**) Log distribution of the node digrees of attack subnetwork.

It can be seen from Figure 6 that the communication relay nodes are in the fourth layer between the C2 nodes, the sensors, and the attack nodes, instead of directly interrupting the sensors, attack nodes, and relay nodes. As long as it is not a formation, the sensors and attack nodes must be connected through relay nodes. Which the attack subnetwork degree distribution is as shown in Figure 7. According to the distribution of node degree of CC,DD, relay nodes can be generated with a certain probability. Only considering the relay nodes [29] of the fourth layer, we increase communication nodes according to the largest connected subgraph and then increase the number of excessively large relay nodes according to the shortest path strategy, which introduces a new kind of scale-free and small-world network with multiple hubs based on classic networks and presents the trapping problem on them. They show that in the multiple-hub networks, the growth of MFPT with the traps located on the hub or peripheral nodes displays quite a difference from the single-hub networks.

Taking the functional of the early warning aircraft as an example, its communication attributes are divided into two parts: internal communication and external communication. The internal communication system establishes voice communication for the operator and the crew; the external communication system consists of several shortwave and ultrashortwave radio stations, which can transmit a large amount of information obtained to the airborne companion aircraft, the sea fleet, or the ground command post. The data transmission can use voice or digital mode. Communication data transmission and cross-linking capabilities are determined by communication capabilities and data transmission capabilities, communication capabilities are determined by air-to-air, air-to-ground, and air–sea communication capabilities, and data transmission capabilities are determined by real time accuracy and reliability [30]. The proposed model not only shows a hierarchically increasing degree of distribution and large clustering coefficients in communities but also exhibits spatial clustering features of individual distributions. As an evaluation of the method, they reconstruct a hierarchical contact network based on the investigation data of a university.

## 3. Dynamic Network Evolution

At present, commonly used attack strategy models include random, deliberate, and incomplete information attack. Among them, incomplete information attacks are closer to real combat. The communication network shows a certain degree of robustness [24] to confront random attacks. The robustness and vulnerability directly affect the effectiveness of the communication network system in combat. Incomplete information attacks can not only reflect the robustness of communication network research and confront random attacks but also indicate the vulnerability of deliberate attacks, which can more truly reflect the changes in the effectiveness of the communication network system in actual operations. At the same time, different from the attack strategy in the previous section, it is more scientific and reasonable to use the incomplete information attack as the attack strategy and the ranking $I_{i}$ of the importance of the node $v_{i}$ in the network as the basis for the basic information of the attacking nodes.

There are a large number of various nodes in the combat SoS, but how to find the most valuable target to attack is significant, in order to achieve the maximum combat effectiveness. In the real combat, the interaction of various information and nodes is becoming more frequent. Simply destroying a node is difficult to cause greater impact and damage to the enemy’s combat system. Therefore, it is necessary to determine the topological structure of the opponent’s combat system and attack the key node to achieve a more ideal effect.

Determination Ω involves two issues: in one, Ω nodes are included in the accuracy of the attack information; the other is the number of Ω nodes contained in and the extent of the attack information. Therefore, the selection of nodes for incomplete information attacks can be compared to the “unequal probability sampling problem” without replacement. Here, α is used to represent the accuracy of the attack information, and β is used to represent the breadth of the attack information and the sample size n=N•β, where β∈(0,1).

The auxiliary variables for constructing nodes $v_{i}$ are as follows:

(2) $$\pi_{i}=r_{i}^{-\alpha }$$

In Formula (2) α∈[0,∞), the sampling (n=1) probability of a simple sampling node $v_{i}$ is:

(3) $$P_{i}=\frac{\pi_{i}}{\sum_{t=1}^{N}\pi_{i}}=\frac{r_{i}^{-\alpha }}{\sum_{t=1}^{N}r_{t}^{-\alpha }}$$

Obviously, the larger the α value is, the greater the possibility that important node information will be obtained, and the higher the accuracy of the attack.

**Figure 8:**
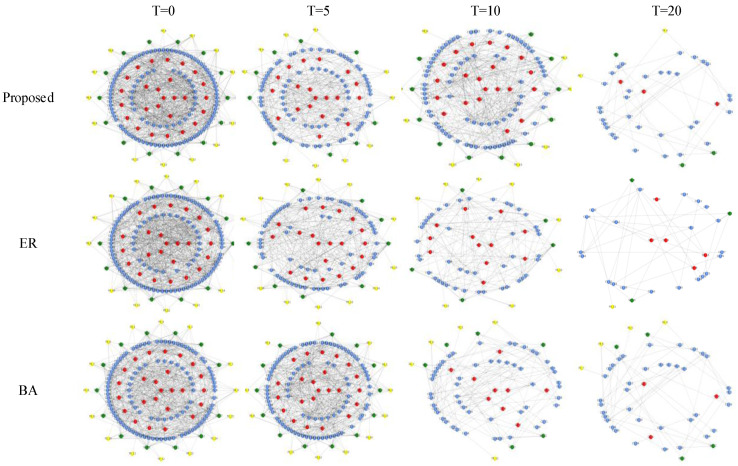
Controlled experimental random network dynamic attack evolution diagram of combat system in continuous time series.

In the pre-war planning (operational planning)–dynamic deployment process, different communication topologies have a greater impact on the survivability, transmission, and repair capabilities of the combat network. Through the dynamic evolution of the combat network under different communication topologies, the study of the change rule of the effectiveness evaluation index of the communication network system of different topologies can evaluate the advantages and disadvantages of the communication topologies. The dynamic evolution of the combat network under continuous time series is shown in Figure 8. As a comparison, we use the network evolution diagram of the ER network after being attacked as a control experiment and select relevant indicators as a comparison description.

## 4. Results and Discussion

It can be seen from Figure 8 that when the combat SoS constructed in this article is hit by incomplete information, the network has a cascading effect. It can be seen that attacking a small part of the key nodes can cause drastic changes in the entire combat network, especially the failure of the central node, leading to the rapid collapse of the lower-level nodes connected to it. At the same time, for communication nodes with command spans, as the number increases, after the hub node connected to the central node is destroyed, a cascade failure [31] occurs in the combat network.

In order to verify that the proposed algorithm is closer to reality, we did the following experiments. In order to reduce the randomness of the experiment, we conducted 30 sets of experiments for each group and took the average value and error analysis to describe the following experimental results.

### 4.1. The Influence of Different Distributed Combat Networks on Various Indicators

**Figure 9:**
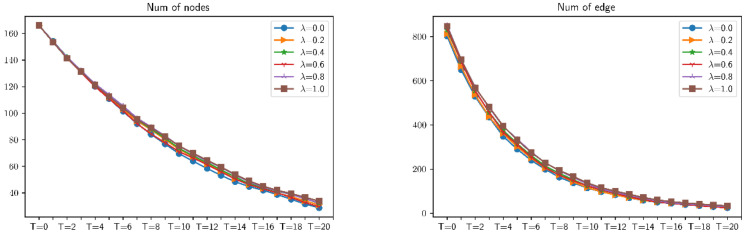
Changes in the number of nodes and edges of differently distributed combat networks under *α* = 0.2, *β* = 0.5 attack.

**Figure 10:**
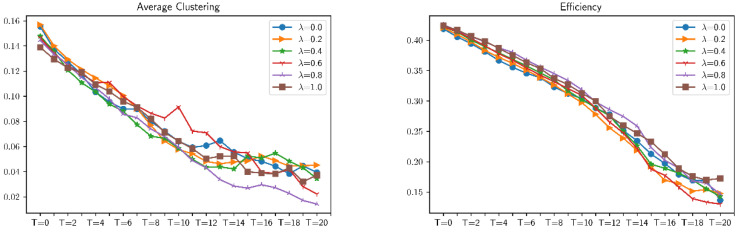

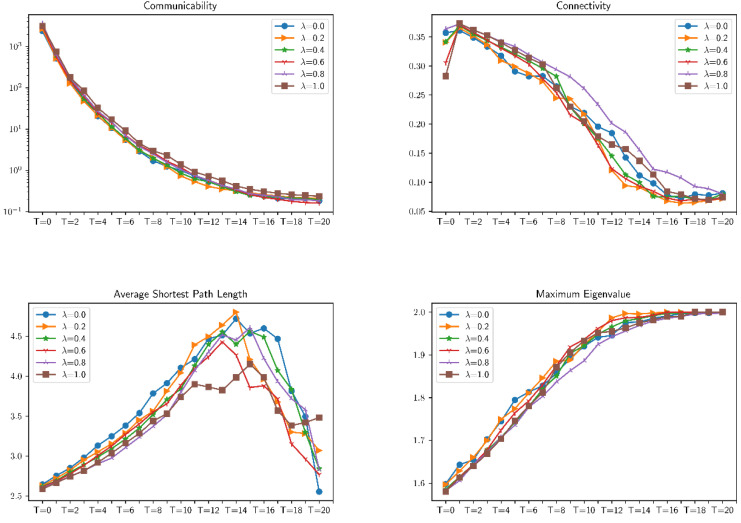
Changes in the robustness of differently distributed combat networks under *α* = 0.2, *β* = 0.5 attack.

This section uses random attacks based on different distributed combat networks to simulate these distributed networks. First of all, for the simulation analysis of six ratios of combat networks in the same attack mode, select six values of λ (respectively 0.0, 0.2, 0.4, 0.6, 0.8, and 1.0), and analyze the effects of different ratios of combat networks under these six values of λ. After the attack, the average clustering, average shortest path length, communication, connectivity, number of edges, number of nodes, efficiency, and maximum eigenvalue have a total of eight indicators affecting the robustness of combat networks. The simulation results are shown in Figure 9 and Figure 10. It can be found that for combat networks with different proportions of ER and BA, under the same attack mode, the changes in indicators are not obvious. In other words, the robustness and vulnerability of different distributed combat networks to the same attack mode are roughly the same.

**Figure 11:**
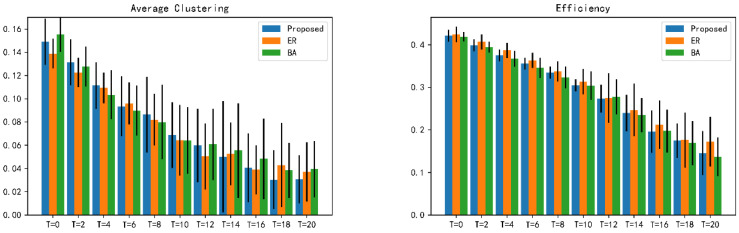

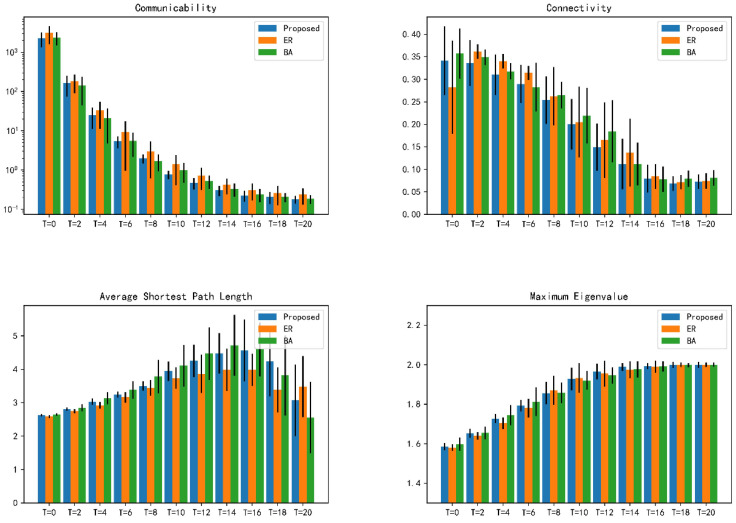
Error analysis of robustness of differently distributed combat networks under *α* = 0.2, *β* = 0.5 attack.

In order to more intuitively show the degree of influence of the same attack method on different distributed combat networks, the following error analysis is used to simulate and verify different distributed combat networks. The error analysis of robustness is shown in Figure 11.

### 4.2. The impact of Different Attack Methods on the Combat Network Indicators

(1) Change the precision α with the breadth β fixing

**Figure 12:**
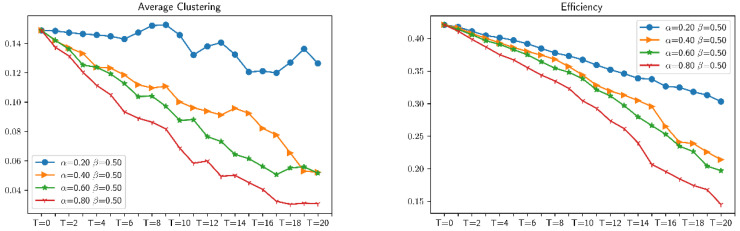

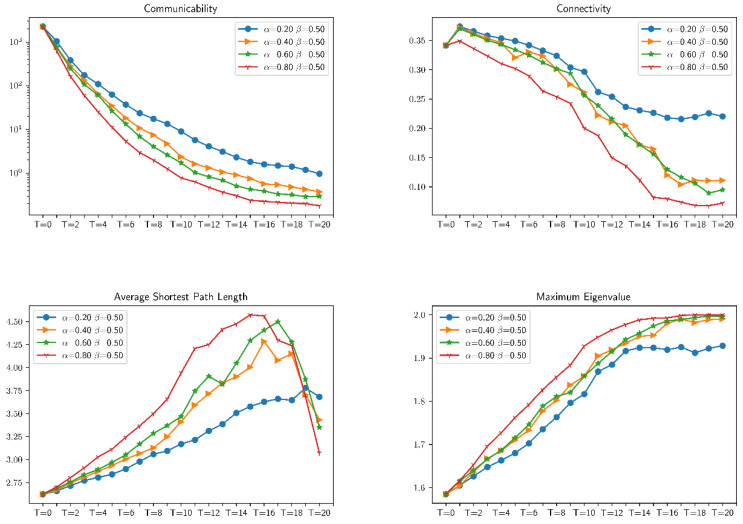
Changes in various indicators of the same distributed combat network under *β* = 0.5.

With the growth of α, the more likely the information of important nodes in the network will be obtained, and the higher the accuracy of the attack. It can be seen from Figure 12 that when α increases, indicators 1–4 show a downward trend. The larger the value of α, the faster the downward trend. There is a positive correlation between α and the magnitude of the downward trend. The average shortest length tends to increase with the α increase. This is because the number of important nodes being attacked continues to increase when the network is continuously attacked. As more and more nodes in the network increasing, the connection needs to rely on more node relays, which causes the path length of the connection between nodes increasing with deeper attacking. There are fewer and fewer important nodes remaining in the combat network, and the remaining edge node distances have a connection relationship due to their own existence, and the entire average shortest length shows a decreasing trend.

(2) Change the breadth β with the precision α fixing

**Figure 13:**
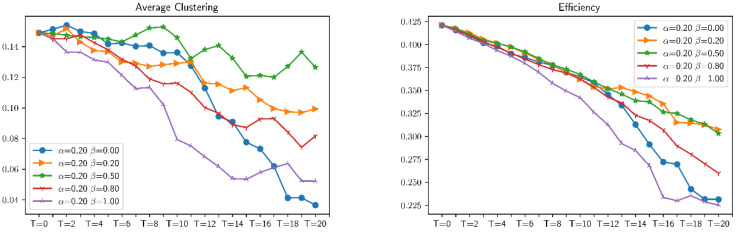

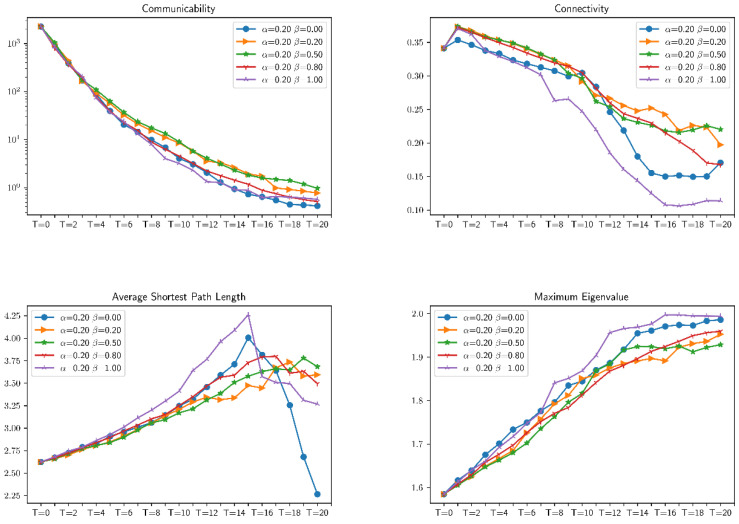
Changes in the indicators of the same distributed combat network under *α* = 0.2.

Similarly, as shown in Figure 13, as the proportion of β increases, the various indicators of the combat network show a downward trend, and the rate of decline depends on the speed of the network collapse. Before the communication subnetwork completely collapses, the larger the β value, the larger the downward trend, and the higher the robustness of the network.

In the same way, we can analyze the trends of several other indicators, and we can see when β=1.0, the combat network will not completely collapse. Through the horizontal comparison of different indicators, it can be found that the constructed network model is more in line with the changing trend of the robustness of the combat system in the actual fighting.

## 5. Conclusions

Communication nodes are particularly important in combat systems. They play an important role in the transmission of command flow, energy flow, and material flow. Attacks on the enemy’s important communication nodes can often produce better effects than other nodes. Therefore, the confrontation scenario can make full use of this feature and have a multiplier effect on combat effectiveness. At the same time, it also provides a basis and reference for the protection and camouflage of communication nodes in the confrontation scenario by determining which nodes and hubs should be protected and which nodes should be repaired first so that the combat effectiveness of the combat system can be maximized.

The communication network is a small-world network composed of an outer layer and an inner layer with a core structure. The outer nodes tend to connect more important nodes. At the same time, the hierarchical nature of the communication network prevents the conflict of information flow in the entire combat network. In order to improve the efficiency and cost of information circulation, the degree distribution of the communication network is a mixed distribution. Compared with the ordinary randomly distributed ER network and the power-law distributed BA network, it has stronger robustness and survivability, and it is also more realistic. Reflecting the communication network topology in the fighting, it can also provide a basis and reference for the calculation of combat effectiveness of the next combat system. On the basis of modeling and simulation, quantitative analysis of combat effectiveness at a certain moment.

## Data Availability

No data were used to support this study.

## Appendix A

**Table A1:** Node in the C2 subnetwork.

No.	Node Name	C2 Level	Quantity	First-Level Attribute	Second-Level Attribute
D0-0	War Zone Joint Command Center	1	1	D	C
D1-0	Ground Command Center	2	1	D	C
D1-1	Air Force and Defence Command Center	2	1	D	C
D1-2	Sea Command Center	2	1	D	C
D1-3	Conventional missile Command Center	2	1	D	C
D1-4	Campaign reserve	2	1	D	C
D2 0-2	Air Force Echelon	3	3	D	C
D2 3-5	Air Defense Regiment	3	3	D	C
D2 6-8	Rader Station and Electronic Warfare Regiment	3	3	D	C
D3 0-16	Task formation	4	17	D	C

**Table A2:** Nodes in the attack and sensors subnetwork.

No.	Node Name	C2 Level	Quantity	First-Level Attribute	Second-Level Attribute
F-1	Air Patrol Formation 1	1	1	F	C
F-2	Air Patrol Formation 2	1	1	F	C
F-3	Air Attack Formation 1 (on the sea)	1	1	F	C
F-4	Air Attack Formation 2 (on land)	1	1	F	C
S-1	Air Attack Formation 3 (counter-submarine)	1	1	S	C D
F-5	Sea-to-Ship Assault Formation 1	1	1	F	C D
F-6	Sea-to-Ship Assault Formation 2	1	1	F	C D
S-2	Sea-to-Ship Assault Formation 3	1	1	S	C
F-7	Force Projection Formation 1	1	1	F	C
F-8	Force Projection Formation 2	1	1	F	C
F-9	Force Projection Formation 3	1	1	F	C
F-10	Force Projection Formation 4	1	1	F	C
F-11	Underwater Attack Team 1 (on land)	1	1	F	S C
F-12	Underwater Attack Team 2 (against the sea)	1	1	F	S C
F-13	Land Attack Formation 1	1	1	F	S C
F-14	Anti-Ship Attack Formation 1	1	1	F	S C
F-15	Air Defense Attack Formation 1	1	1	F	S C

**Table A3:** Nodes in the communication subnetwork.

No.	Node Name	C2 Level	Quantity	First-Level Attribute	Second-Level Attribute
C	Communication assurance	…	113	C	…
